# Type 2 Diabetes and Cognitive Status in the Health and Retirement Study: A Mendelian Randomization Approach

**DOI:** 10.3389/fgene.2021.634767

**Published:** 2021-03-25

**Authors:** Erin B. Ware, Cristina Morataya, Mingzhou Fu, Kelly M. Bakulski

**Affiliations:** ^1^Population Neurodevelopment and Genetics, Survey Research Center, Institute for Social Research, University of Michigan, Ann Arbor, MI, United States; ^2^Population Studies Center, Institute for Social Research, University of Michigan, Ann Arbor, MI, United States; ^3^Department of Epidemiology, School of Public Health, University of Michigan, Ann Arbor, MI, United States

**Keywords:** type 2 diabetes mellitus, dementia, polygenic score, health and retirement study (HRS), Mendelian randomization

## Abstract

**Background:**

Type 2 diabetes mellitus (T2DM) and dementia are leading causes of mortality and disability in the US. T2DM has been associated with dementia; however, causality has not been clearly established. This study tested inferred causality between T2DM and dementia status using a Mendelian randomization approach.

**Methods:**

Participants (50+ years) from the 2010 wave of the Health and Retirement Study of European or African genetic ancestry were included (*n* = 10,322). History of T2DM was self-reported. Cognitive status (dementia, cognitive impairment non-dementia, or normal cognition) was defined from clinically validated cognitive assessments. Cumulative genetic risk for T2DM was determined using a polygenic score calculated from a European ancestry T2DM genome-wide association study by Xue et al. (2018). All models were adjusted for age, sex, education, *APOE*-ε4 carrier status, and genetic principal components. Multivariable logistic regression was used to test the association between cumulative genetic risk for T2DM and cognitive status. To test inferred causality using Mendelian randomization, we used the inverse variance method.

**Results:**

Among included participants, 20.9% had T2DM and 20.7% had dementia or cognitive impairment. Among European ancestry participants, T2DM was associated with 1.66 times odds of cognitive impairment non-dementia (95% confidence interval: 1.55–1.77) relative to normal cognition. A one standard deviation increase in cumulative genetic risk for T2DM was associated with 1.30 times higher odds of T2DM (95% confidence interval: 1.10–1.52). Cumulative genetic risk for T2DM was not associated with dementia status or cognitive-impaired non-dementia in either ancestry (*P* > 0.05); lack of association here is an important assumption of Mendelian randomization. Using Mendelian randomization, we did not observe evidence for an inferred causal association between T2DM and cognitive impairment (odds ratio: 1.04; 95% confidence interval: 0.90–1.21).

**Discussion:**

Consistent with prior research, T2DM was associated with cognitive status. Prevention of T2DM and cognitive decline are both critical for public health, however, this study does not provide evidence that T2DM is causally related to impaired cognition. Additional studies in other ancestries, larger sample sizes, and longitudinal studies are needed to confirm these results.

## Background

Type 2 diabetes mellitus (T2DM) is robustly associated with dementia. Multiple studies have found a positive association between T2DM and cognitive impairment (Hassing et al., 2004; Baumgart et al., 2015; Østergaard et al., 2015; Cholerton et al., 2016). A meta-analysis study reported a 73% increased risk of all-type dementia in diabetes patients (Gudala et al., 2013). While there are several potential mechanisms to support such findings (Gasparini et al., 2001; Townsend et al., 2007), it is unclear whether and how these two conditions are causally linked. It is also possible that the direction of causality might actually be the reverse; that is, dementia causes T2DM. Both T2DM and dementia exert enormous burdens on individuals and on healthcare systems, especially given that there is a dramatic rise in prevalence and there is currently no cure for either disease. Further, previous studies have observed higher plasma glucose concentrations in those with unspecified dementia compared to those with Alzheimer’s disease and vascular dementia (Benn et al., 2020). In the presence of T2DM, those with unspecified dementia were less often treated with oral glucose-lowering drugs. This suggests a contributing factor to dementia risk may stem from poor glycemic control and improving diabetes management and diagnosis may help alleviate the burden of dementia. Thus, it is extremely important to understand the underlying relationships between T2DM and dementia.

Mendelian randomization is an advanced approach for estimating causal effects between risk factors with genetic determinants. It is predicated on the random assortment of genes at meiosis, which is akin to a “genetically randomized trial,” so that the results are generally independent of the environmental confounders and less subject to reverse causation (Davey Smith and Hemani, 2014). T2DM is a complex disease which involves polygenic etiologic contributing factors (Hao et al., 2015). According to a recent genome-wide association study (GWAS), over 65 susceptibility genes have been identified, which totally explained ∼63% of the inter-individual variation of T2DMsusceptibility (Morris et al., 2012). With this large genetic component, genetic predisposition to T2DM can be used as an instrumental variable to infer causality of T2DM on cognitive impairment in a Mendelian randomization framework.

In our current study, we sought to estimate the associations and causal effects of T2DM on dementia using Mendelian randomization in a United States nationally representative aging cohort. The aim of this study is to inform the etiology of dementia and the extent to which cognitive impairment could be preventable by interventions targeting a potentially modifiable risk factor, T2DM.

## Materials and Methods

### Study Population From the Health and Retirement Study

We used a cross-sectional sample of participants from the 2010 wave of the Health and Retirement Study (HRS), a nationally representative longitudinal panel study of persons aged 50 years and older, funded by the National Institute on Aging (NIA U01AG009740) and the Social Security Administration (SGIM, 2019). This study was approved by the University of Michigan Institutional Review Board HUM00128220. We selected the 2010 wave for this study due to its inclusion of a broad range of birth cohorts, availability of self-reported history of diabetes, distribution of cognition classifications, and availability of genetic data collected in 2006, 2008, and 2010.

Sample selection steps are shown in Supplementary Figure 1. We excluded participants who were younger than 50 or over 90 at wave 2010 because the underlying neuropathological mechanisms and risk factors of dementia are considerably different in those age groups (Bullain and Corrada, 2013). Given the irreversibility of cognition decline, we also excluded individuals who reported a dementia measure in the prior wave (wave 2008) and normal cognition in the current 2010 wave to minimize misclassification of cognitive status.

### Outcome, Exposure Assessments, and Demographic Characteristics

HRS conducts measurements of cognition using a series of cognitive tests including immediate and delayed word recall, serial 7 s subtraction, and backward counting from 20. Respondents who could not answer for themselves due to physical or mental disability at the interview, were evaluated by a proxy based on their performances on memory, five instrumental activities of daily living (using a telephone, taking medication, handling money, shopping, and preparing meals), and difficulty completing interview because of cognitive limitation (Jorm, 1994). Our main outcome, the Langa-Weir cognitive status, was classified in three levels based on a total score of 27-point for self-respondents(normal: 12–27, cognitive impairment-non dementia (CIND): 7–11, and dementia: 0–6); and 11-point for proxy-respondents (normal: 0–2, CIND: 3–5, and dementia: 6–11). The Langa-Weir approach has been clinically validated with an area under curve score of 0.84 (Crimmins et al., 2011).

History of T2DM (yes/no) was self-reported in response to “has a doctor ever told you that you have diabetes or high blood sugar?”Other covariates used in our analysis included demographic characteristics, behavioral risk factors, and chronic health conditions. Age in 2010 (years) was calculated from self-reported year of birth. Sex (male/female), years of education, proxy status (self/proxy-respondent), body mass index (BMI, kg/m^2^), smoking status (never/former/current), alcohol consumption (ever/never), history of hypertension or stroke(yes/no) were self-reported. All variables were assessed at the 2010 wave and retrieved from the RAND HRS Longitudinal File 2016 (V2), produced by the RAND Center for the Study of Aging (Rand, 2019).

### Genetic Data

Since 2006, HRS has collected genetic data during an enhanced face-to-face interview after consenting respondents. Details of the genotype collection and quality control can be found elsewhere (David, 2013). Around 2.5 million single nucleotide polymorphisms (SNPs) were genotyped using the Illumina HumanOmni2.5 BeadChip (HumanOmni2.5–4v1, HumanOmni2.5–8v1). Genotype data that passed initial quality control were released and analyzed by the Quality Assurance/Quality Control analysis team at the University of Washington. All genetic data are available from the National Center for Biotechnology Information’s database of genotypes and phenotypes (dbGaP Study Accession: phs000428.v2.p2).

Genetic ancestry was assigned based on consistent results from self-reported race/ethnicity and principal component (PC) analysis on genome-wide SNPs. By HRS data release, data were not available on participants with mixed genetic ancestry or participants with differences in self-reported race/ethnicity and genetic ancestry. Within each ancestry sample, another PC analysis was conducted to create ancestry-specific PCs which aim at adjusting for hidden population structure within ancestry. In the HRS, *APOE* gene carrier status were categorized as ε2/ε2, ε2/ε3, ε2/ε4, ε3/ε3, ε3/ε4, and ε4/ε4 using genetic data imputed to the 1000 Genomes Project reference panel (phase I) (The 1000 Genomes Project Consortium, 2010). An indicator variable of the presence of any copy of *APOE*-*ε4* allele (yes/no) was used in our primary analyses. All genetic data were downloaded from published datasets by the HRS (Health and Retirement Study, 2020).

Polygenic scores were used as instrumental variables in our Mendelian randomization analyses. A polygenic score is a summary score of risk variants weighted by effect estimates from a GWAS that broadly represents a subject’s cumulative genetic risk of a phenotype of interest (Ware et al., 2019). We created polygenic scores for T2DM and Alzheimer’s disease from the HRS genotyped data from 2006 through 2010, following the exact polygenic score pipeline that the HRS releases (Ware et al., 2017a). We calculated our own polygenic scores because we were interested in examining different GWAS *P*-value cutoff thresholds for inclusion in the scores and removing the *APOE* region (chr19:45, 384,477–45,432,606, build 37/hg 19) from the Alzheimer’s disease polygenic score (Ware et al., 2020). We calculated polygenic scores using all SNPs which overlapped between the HRS genetic database and the GWAS meta-analysis of interest. Polygenic scores (PGS) were calculated as follows: $P\,G\,S_{i}=\sum_{j=1}^{J}W_{j}\,G_{i\,j}$, where i corresponds to individual i, j is SNP j (*j* = 1–*J*), W is the meta-analysis effect size for SNP j, and G is the genotype, or the number of reference alleles (zero, one, or two), for individual i at SNP j (Ware et al., 2019). Effect sizes for T2DM were obtained from a 2018 European ancestry GWAS meta-analysis conducted by Xue et al. (2018), and effect sizes for AD were from a 2019 GWAS by Kunkle et al. (2019) excluding the *APOE* region (Ware et al., 2020). As suggested by prior research in this sample, we selected a more conservative *p*-value threshold of 0.01 for the Alzheimer’s disease polygenic score as this cutoff was shown to be more appropriate than a *p*-value threshold of 1 (Ware et al., 2020). All the polygenic scores were standardized to a standard normal curve (mean = 0, standard deviation = 1) within ancestry.

### Statistical Analysis

We compared distributions of baseline characteristics between included and excluded samples, European and African ancestry samples, and across exposure (history of T2DM) and outcome (cognitive status) groups. Homogeneity across groups was tested using χ^2^-test or analysis of variance as appropriate.

**FIGURE 1:**
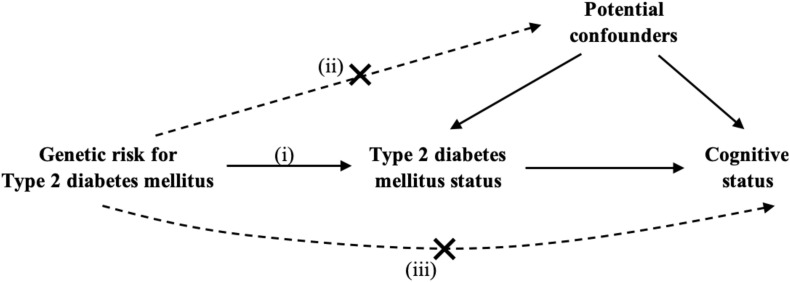
Mendelian randomization analyses structure and subsets of study population, Health and Retirement Study, Wave 2010 (*n* = 10,322). CIND, cognitive impairment non-dementia. ^a^Mendelian randomization assumptions: (i) the genetic variant must be associated with the exposure, (ii) the genetic variant must not be associated with any confounder of the exposure-outcome association and (iii) the genetic variant must be associated with the outcome only via the exposure. ^b^Subsets used in multivariable logistic regression and Mendelian randomization analyses: participants with (A) non-missing values in Type 2 diabetes mellitus status + CIND or normal cognitive status; (B) non-missing values in Type 2 diabetes mellitus status + dementia or normal cognitive status.

Our main analyses were in the European ancestry sample. The assumption of proportional odds for ordinal logistic regressions were violated so we performed multivariable logistic regressions and Mendelian randomization in two subsets of each ancestry sample. First, using the cognitively impaired non-dementia and normal cognitive status and second, the dementia and normal cognitive status groups. Normal cognition and no T2DMwere considered the reference group(s). Figure 1 shows the heuristic model and study subsets.

We conducted aninverse variance weighted Mendelian randomization analysis, using a polygenic score for T2DM which included all available SNPs (*P* threshold = 1) that overlap between the GWAS and the HRS genetic data to increase the power. Different *P* thresholds (0.3, 0.1, 0.05, 0.01, and 0.001) for the T2DM polygenic score were also explored in sensitivity models to check the robustness of our findings. Wald-type ratio estimator were used to infer causality and the standard errors were estimated using the Delta method (Davies et al., 2018). Results from the multivariable logistic regression and Mendelian randomization were compared using test of interaction to evaluate heterogeneity (Altman and Bland, 2003).

Alzheimer’s disease (AD) is the most common type of dementia, which accounts for up to 80% of dementia cases (Alzheimers Association, 2020). Similar to T2DM, AD also involves multiple etiologic contributing factors, among which genetic predisposition factors, such as *APOE*, are known to play important roles (Hao et al., 2015). Thus, we also took genetic risks of AD into consideration in our analysis. Our primary models were adjusted for age, sex, years of education, *APOE*-*ε4* allele status, and five ancestry-specific PCs. Additional adjustment included health risk factors that were associated with T2DM and cognitive status(i.e., history of stroke, hypertension, BMI, smoking and drinking status),as well as an Alzheimer’s disease polygenic score(*P* threshold = 0.01) with the *APOE* gene region removed (Ware et al., 2020).

We tested the three assumptions of Mendelian randomization to assess the robustness of our findings. Improvement χ^2^ was used to evaluate the relevance assumption, that the genetic variant must be associated with the exposure; and values greater than 10 were taken as evidence against weak instruments (Davies et al., 2018). To tentatively check independence and exclusion restriction assumptions, we examined the associations between the T2DM polygenic score and potential confounding factors using linear regressions and calculated Pearson correlations between the Alzheimer’s disease and T2DM polygenic scores. We also used a subset of individuals withoutT2DM as a negative control, in which there should be no association between the T2DM polygenic score and cognition status if the assumptions hold.

In the sensitivity analyses, we used two-sample Mendelian randomization methods with published summary statistics from GWASs to further test the robustness of our results. Multiple recently developed methods including Mendelian randomization-Egger and weighted median methods. The two-sample Mendelian randomization analysis was conducted using the *TwoSampleMR* package (Hemani et al., 2019)Using the summary statistics from the two same GWASs (T2DM and AD Kunkel), we analyzed the causal relationship between T2DM and AD with all overlapped SNPs (significant threshold = 1), without LD clumping to maintain consistency with our main analysis. We repeated our analyses in participants between 50 and 80 years only to account for potential mortality selection bias. We also ran a sensitivity analysis within an older group (age 65–90) to exclude participants who may be too young to develop dementia. We additionally tested reversed causality (dementia on T2DM) using the Alzheimer’s disease polygenic score (*P* threshold = 0.01) as an instrumental variable in a similar Mendelian randomization framework. Similar steps were also performed to check if the three assumptions met. Finally, all analyses were additionally conducted in the African ancestry sample.

All analyses were performed in R statistical software (version 3.6.1) (R Foundation, 2019). We reported odds ratios (OR) for logistic regression and β coefficients for linear regression along with their 95% confidence intervals (CIs). *P* < 0.05 was considered as statistical significance if not specified. All analyses were carried out separately by genetic ancestry and adjusted for a set of five ancestry-specific PCs to adjust for population stratification within ancestry group. Population attributable fractions were also calculated for significant associations. Code to produce all analyses in this manuscript are available online^1^.

## Results

A total of 10,322 participants from the 2010 wave of the HRS were eligible for our final study sample. Compared to excluded participants, the included participants were older, more educated, with better cognition and less likely to have T2DM (Supplementary Table 1). The included European ancestry sample (*n* = 8,433) were older, more educated, had a lower prevalence of the *APOE*-*ε4* allele, better cognition and were less likely to have chronic health conditions, including T2DM, than the included African ancestry sample (*n* = 1,889) (Supplementary Table 2).

In the European ancestry sample, age, sex, educational attainment, smoking status, alcohol consumption, stroke, and hypertension status, and BMI, were associated with both history of T2DM and cognitive status. These variables were adjusted as confounders in the following analyses (Table 1). Similar associations were also found in the African ancestry sample (Supplementary Table 3).

**TABLE 1:** Bivariate characteristics stratified by cognitive status or history of Type 2 diabetes mellitus, Health and Retirement Study, Wave 2010, European ancestry sample (*n* = 8,433)^a^.

	**Cognitive status**					**Type 2 diabetes mellitus status**			
	**Overall**	**Normal**	**CIND**	**Dementia**	***P*-value^b^**	**Overall**	**Yes**	**No**	***P*-value^b^**
	***n* = 8,433**	***n* = 6,995**	***n* = 1,119**	***n* = 319**		***n* = 8,433**	***n* = 1,601**	***n* = 6,832**	
History of Type 2 diabetes mellitus (Yes)	1,601 (19.0%)	1,242 (17.8%)	282 (25.2%)	77 (24.1%)	<0.001*	–			
Type 2 diabetes mellitus polygenic score^c^	0.00 (1.00)	0.00 (1.01)	0.00 (0.95)	0.02 (0.97)	0.94	0.00 (1.00)	0.33 (0.97)	−0.07 (0.99)	<0.001*
Alzheimer’s disease polygenic score^d^	0.00 (0.99)	−0.01 (0.99)	0.02 (1.01)	0.14 (1.03)	0.03*	0.00 (0.99)	0.00 (0.99)	0.00 (1.00)	0.90
*APOE-ε4* allele carrier (Yes)	2,233 (26.5%)	1,766 (25.2%)	335 (29.9%)	132 (41.4%)	<0.001*	2,233 (26.5%)	410 (25.6%)	1,823 (26.7%)	0.40
Sex (Female)	4,860 (57.6%)	4,104 (58.7%)	583 (52.1%)	173 (54.2%)	<0.001*	4,860 (57.6%)	808 (50.5%)	4,052 (59.3%)	<0.001*
Stroke history (Yes)	611 (7.25%)	367 (5.25%)	143 (12.8%)	101 (31.8%)	<0.001*	611 (7.25%)	179 (11.2%)	432 (6.33%)	<0.001*
Hypertension history (Yes)	4,874 (57.9%)	3,910 (56.0%)	732 (65.4%)	232 (73.4%)	<0.001*	4,874 (57.9%)	1,270 (79.3%)	3,604 (52.8%)	<0.001*
Smoking status					<0.001*				<0.001*
Never	3,647 (43.5%)	3,078 (44.3%)	431 (38.7%)	138 (43.3%)		3,647 (43.5%)	671 (42.1%)	2,976 (43.8%)	
Former	3,768 (44.9%)	3,078 (44.3%)	532 (47.8%)	158 (49.5%)		3,768 (44.9%)	773 (48.6%)	2,995 (44.1%)	
Current	969 (11.6%)	795 (11.4%)	151 (13.6%)	23 (7.21%)		969 (11.6%)	148 (9.30%)	821 (12.1%)	
Drink status (Ever drinker)	4,782 (56.7%)	4,252 (60.8%)	463 (41.4%)	67 (21.0%)	<0.001*	4,782 (56.7%)	709 (44.3%)	4,073 (59.6%)	<0.001*
Age at 2010 (years)	69.6 (10.1)	68.2 (9.59)	75.4 (9.67)	80.3 (7.31)	<0.001*	69.6 (10.1)	71.0 (9.39)	69.2 (10.2)	<0.001*
Years of education	13.3 (2.49)	13.6 (2.34)	12.0 (2.68)	11.7 (2.91)	<0.001*	13.3 (2.49)	12.9 (2.52)	13.4 (2.48)	<0.001*
Body mass index (kg/m^2^)	28.0 (5.79)	28.2 (5.75)	27.3 (5.96)	25.2 (4.99)	<0.001*	28.0 (5.79)	30.9 (6.49)	27.3 (5.39)	<0.001*

*APOE, Apolipoprotein E; CIND, cognitive impairment non-dementia; SD, standard deviation; T2DM, Type 2 diabetes mellitus.*

*^*a*^All the statistics including count, frequency, mean, standard deviation, and P-value were calculated based on non-missing data for each variable.*

*^*b*^The P-value was calculated from chi-square test or analysis of variance for categorical or continuous variables as appropriate, interpreted as differences between groups.*

*The asterisk indicates statistical significance at 0.05 level.*

*^*c*^The Type 2 diabetes mellitus polygenic score was created using weights from a genome-wide association study meta-analysis from the DIAbetes Genetics Replication and Meta-analysis Consortium (Morris et al., 2012) using a P-value threshold of 1.*

*^*d*^The Alzheimer’s disease polygenic score was created using weights from a genome-wide association study meta-analysis from a 2019 GWAS by Kunkle et al. (2019)*using a P-value threshold of 0.01 and removing the APOE region (chr19: 45,384,477–45,432,606, build 37/hg 19)* (Ware et al., 2020).*

### Associations With the T2DM Polygenic Score

The T2DM polygenic score was positively associated with history of T2DM in the European ancestry sample (Table 2). A one standard deviation increase in the T2DM polygenic score was associated with 1.66 (95% CI: 1.55, 1.77) times higher odds of T2DM, adjusting for age, sex, years of education, *APOE*-*ε4* allele status, and five ancestry-specific PCs. Formal test statistics confirmed the T2DM polygenic score as a valid instrument for history of T2DM (improvement χ^2^: 253.7 > 10, Table 2).

**TABLE 2:** Associations between polygenic score for Type 2 diabetes mellitus and Type 2 diabetes mellitus status, Health and Retirement Study, Wave 2010, European ancestry sample (*n* = 8,289)^a^.

	**Overall sample, *n* = 8,289**		**CIND and normal cognition sample, *n* = 7,979**		**Dementia and normal cognition sample, *n* = 7,186**	
	**OR**	**(95 CI%)**	**OR**	**(95 CI%)**	**OR**	**(95 CI%)**
Crude^b^	1.54	(1.45, 1.63)	1.52	(1.44, 1.62)	1.55	(1.46, 1.65)
Adjusted^c^	1.66	(1.55, 1.77)	1.64	(1.54, 1.75)	1.69	(1.58, 1.82)
**Improvement χ^2d^**	253.7		234.1		232.7	

*CI, confidence interval; CIND, cognitive impairment-non dementia; OR, odds ratio.*

*^*a*^All the values were based on results from multivariable logistic regression analyses in each sample, in which “no diabetes history” was used as the reference group.*

*^*b*^The Type 2 diabetes mellitus polygenic score was created using weights from a genome-wide association study meta-analysis from the DIAbetes Genetics Replication and Meta-analysis Consortium (Morris et al., 2012) using a P-value threshold of 1.*

*^*c*^Adjusted for age, sex, years of education, APOE-ε4 allele status, and five ancestry-specific principal component sets.*

*^*d*^Calculated by 2^∗^(log likelihood of full model − log likelihood of reduced model). By convention, statistics larger than 10 indicate a valid instrument.*

**FIGURE 2:**
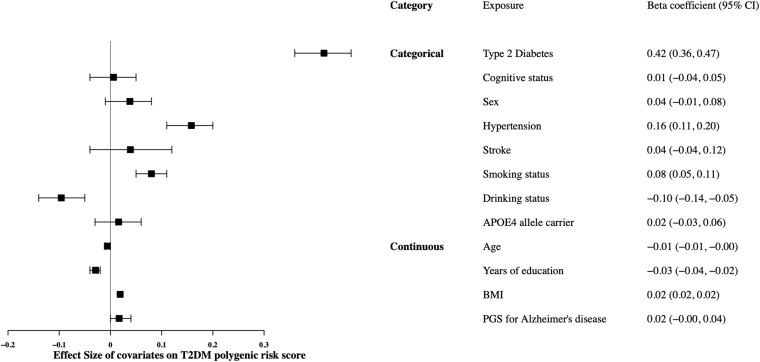
Associations between polygenic score for Type 2 diabetes mellitus^a^ and factors potentially confounding the relation between Type 2 diabetes mellitus and cognitive status, Health and Retirement Study, Wave 2010, European ancestry sample (*n* = 8,433). APOE, Apolipoprotein E; BMI, Body Mass Index; CI, Confidence Interval; PGS, polygenic score. ^a^The Type 2 diabetes mellitus polygenic score was created using weights from a genome-wide association study meta-analysis from the DIAbetes Genetics Replication and Meta-analysis Consortium (Morris et al., 2012) using a *P*-value threshold of 1. ^b^The cognitive status was ordered as normal-CIND-dementia (0-1-2). ^c^The smoking status was ordered as never-former-current (0-1-2). ^d^The Alzheimer’s disease polygenic score was created using weights from a genome-wide association study meta-analysis from a 2019 GWAS by Kunkle et al. (2019) using a *P*-value threshold of 0.01 and removing the *APOE* region (chr19: 45,384,477–45,432,606, build 37/hg 19) (Ware et al., 2020).

In the European ancestry sample, the T2DM polygenic score was not associated with impaired cognition, either CIND or dementia. The null associations remained after further adjustment of history of T2DM (Table 3). Figure 2 presents the associations of the T2DM polygenic score with baseline characteristics in the European ancestry. The T2DM polygenic score was unrelated to all potential confounding factors except for history of hypertension, education, and BMI, indicating a potential violation of the independence assumption of Mendelian randomization.

**TABLE 3:** Associations between polygenic score for Type 2 diabetes mellitus and cognitive status, Health and Retirement Study, Wave 2010, European ancestry sample (*n* = 8,433)^a^.

	**CIND vs. normal, *n* = 7,979**		**Dementia vs. normal, *n* = 7,186**	
	**OR**	**(95 CI%)**	**OR**	**(95 CI%)**
**Total effect of T2DM polygenic score^b^**				
Crude	1.00	(0.94, 1.06)	1.02	(0.91, 1.15)
Adjusted (primary)^c^	1.02	(0.95, 1.10)	1.11	(0.97, 1.27)
Adjusted (health status)^d^	1.01	(0.93, 1.08)	1.09	(0.94, 1.25)
Adjusted (AD genetics)^e^	1.01	(0.93, 1.08)	1.09	(0.94, 1.25)
**Direct effect of T2DM polygenic score (adjusting for history of T2DM)**				
Crude	0.97	(0.91, 1.03)	0.99	(0.88, 1.11)
Adjusted (primary)	1.00	(0.93, 1.08)	1.08	(0.95, 1.25)
Adjusted (health status)	0.99	(0.92, 1.07)	1.07	(0.92, 1.23)
Adjusted (AD genetics^f^)	0.99	(0.92, 1.07)	1.07	(0.92, 1.23)

*AD, Alzheimer’s disease; APOE, Apolipoprotein E; BMI, body mass index; CI, confidence interval; CIND, cognitive impairment-non dementia; OR, odds ratio; T2DM, Type 2 diabetes mellitus.*

*^*a*^All the values were based on results from multivariable logistic regression analyses in each sample, in which “normal cognitive status” and “no diabetes history” were used as reference groups.*

*^*b*^The Type 2 diabetes mellitus polygenic score was created using weights from a genome-wide association study meta-analysis from the DIAbetes Genetics Replication and Meta-analysis Consortium (Morris et al., 2012) using a P-value threshold of 1.*

*^*c*^Adjusted for age, sex, years of education, APOE-ε4 allele status, and five ancestry-specific principal component sets.*

*^*d*^Adjusted for smoking status, ever drink alcohol, history of stroke, hypertension, and BMI in addition to variables in [c].*

*^*e*^Adjusted for Alzheimer’s disease polygenic score in addition to variables in [d].*

*^*f*^The Alzheimer’s disease polygenic score was created using weights from a genome-wide association study meta-analysis from a 2019 GWAS by Kunkle et al. (2019)*using a P-value threshold of 0.01 and removing the APOE region (chr19: 45,384,477–45,432,606, build 37/hg 19)* (Ware et al., 2020).*

### Associations Between T2DM and Cognitive Status

T2DM was positively associated with cognitive impairment in the European ancestry sample (Table 4). In the primary adjusted model, history of T2DM was associated with 1.30 (95% CI: 1.10, 1.52) times odds of CIND relative to normal cognition. Population attributable fraction analysis indicated that 4.4% of the CIND cases were attributed to T2DM in our sample. This positive association held after additional adjustments of health status and AD genetics. However, no association was observed between history of T2DM and dementia.

**TABLE 4:** Associations between history of Type 2 diabetes mellitus and cognitive status, Health and Retirement Study, Wave 2010, European ancestry sample (*n* = 8,433)^a^.

	**Logistic regression**		**Wald-type/ratio**		***P* for heterogeneity^c^**
	**OR**	**(95 CI%)**	**OR**	**(95 CI%)**	
**CIND vs. normal (n = 7,979)^b^**					
Crude	1.56	(1.34, 1.81)	1.00	(0.86, 1.16)	0.04*
Adjusted (primary)^d^	1.30	(1.10, 1.52)	1.04	(0.90, 1.21)	0.05*
Adjusted (health status)^e^	1.30	(1.09, 1.54)	1.01	(0.87, 1.18)	0.03*
Adjusted (AD genetics)^f^	1.30	(1.09, 1.54)	1.01	(0.87, 1.18)	0.03*
**Dementia vs. normal (n = 7,186)**					
Crude	1.53	(1.17, 1.98)	1.05	(0.81, 1.36)	0.07
Adjusted (primary)	1.28	(0.95, 1.72)	1.21	(0.94, 1.56)	0.90
Adjusted (health status)	1.36	(0.98, 1.87)	1.17	(0.90, 1.53)	0.48
Adjusted (AD genetics)^g^	1.34	(0.97, 1.85)	1.17	(0.90, 1.53)	0.52

*AD, Alzheimer’s disease; APOE, Apolipoprotein E; BMI, body mass index; CI, confidence interval; CIND, cognitive impairment-non dementia; OR, odds ratio.*

*^*a*^All values were based on results from multivariable logistic regression or Mendelian randomization analyses in each sample, in which “normal cognitive status” and “no diabetes history” were used as reference groups.*

*^*b*^The Type 2 diabetes mellitus polygenic score was created using weights from a genome-wide association study meta-analysis from the DIAbetes Genetics Replication and Meta-analysis Consortium (Morris et al., 2012) using a P-value threshold of 1.*

*^*c*^P-value represents the statistical significance of the test of heterogeneity between logistic regression and Mendelian randomization. The asterisk indicates statistical significance at 0.05 level.*

*^*d*^Adjusted for age, sex, years of education, APOE-ε4 allele status, and five ancestry-specific principal component sets.*

*^*e*^Adjusted for smoking status, ever drink alcohol, history of stroke, hypertension, and BMI in addition to variables in [d].*

*^*f*^Adjusted for Alzheimer’s disease polygenic score in addition to variables in [e].*

*^*g*^The Alzheimer’s disease polygenic score was created using weights from a genome-wide association study meta-analysis from a 2019 GWAS by Kunkle et al. (2019) using a P-value threshold of 0.01 and removing the APOE region (chr19: 45,384,477–45,432,606, build 37/hg 19) (Ware et al., 2020).*

In Mendelian randomization analysis, we did not detect evidence for a causal relationship between history of T2DM and cognitive status. The results from Mendelian randomization were different from the multivariable logistic regression model estimates (*P* for heterogeneity < 0.05, Table 4). The T2DM polygenic score was not associated with the Alzheimer’s disease polygenic score in our European ancestry sample (*r* = −0.01, *P* = 0.31), which was against potential violations of pleiotropy. In the subsample of people without T2DM (*n* = 6,711), the T2DM polygenic score was not associated with cognitive status (Supplementary Table 4), which supported the likely hold of the independence assumption of Mendelian randomization.

### Sensitivity Analyses

Similar to our primary findings, no causal relationship was found between history of T2DMon cognitive status using T2DM polygenic scores with different *P* thresholds (0.3, 0.1, 0.05, 0.01, and 0.001) in the European ancestry sample. However, the T2DM polygenic scores with lower *P*-value thresholds showed an increased significance of causality (though not significant at 0.05 level), indicating a potential causal relationship between T2DM and cognitive impairment when ruling out weak variants with lower significance in SNP-T2DM associations (Supplementary Table 5). Based on the two-sample Mendelian randomization analyses, we did not observe a causal relationship between T2DM and AD (MR-Egger: *OR* = 1.00, 95% CI: 0.99, 1.01; weighted-median: *OR* = 1.01, 95% CI: 0.99, 1.02).

Regression results were similar in the younger European population, restricting to participants between ages 60 and 80 (*n* = 6,952) (Supplementary Table 6). The T2DM polygenic score remained significantly associated with history of T2DM (*OR* = 1.65, 95% CI: 1.54, 1.77). In the primary model which adjusted for age, sex, years of education, *APOE*-*ε4* allele status, and five ancestry-specific PCs, T2DM was associated with increased odds of CIND at a similar magnitude (*OR* = 1.31, 95% CI: 1.08, 1.58) to the unrestricted analysis. In the older European ancestry sample (age between 65 and 90, n = 5,479), we observed similar results between T2DM and CIND (*OR* = 1.27, 95% CI: 1.06, 1.52). We also found significant associations between T2DM and dementia, adjusting for age, sex, years of education, *APOE*-*ε4* allele status, five ancestry-specific PCs, smoking status, ever drinking alcohol, history of stroke, hypertension, and BMI (*OR* = 1.41, 95% CI: 1.01, 1.95); as well as in a model further adjusted for AD polygenic score (*OR* = 1.39, 95% CI: 1.00, 1.92). However, no causal relationship was found between history of T2DM and any cognitive status using Mendelian randomization in any of the age selected samples.

A reversed causal relationship was also examined between cognitive status on history of T2DM. Similarly, after adjusting for age, sex, years of education, *APOE*-*ε4* allele status, and five ancestry-specific PCs, CIND relative to normal cognition was associated with an increased odd of T2DM (*OR* = 1.23, 95% CI: 1.05, 1.45); while no association was found between dementia and history of T2DM.No causal relationship was observed between any cognitive status and history of T2DM (Supplementary Table 8, Model 2). However, the Alzheimer’s disease polygenic score was associated with dementia only (*OR* = 1.19, 95% CI: 1.05, 1.35), leaving the relevance assumption only met for the subset of people with dementia or normal cognition (Supplementary Table 8, Model 1). Other assumptions were also checked and showed limited evidence of potential violations.

In the African ancestry sample (*n* = 1,889), the T2DM polygenic score was positively associated with history of T2DM (*OR* = 1.25, 95% CI: 1.10, 1.41). Improvement χ^2^ results showed the T2DM polygenic score as a valid instrument for history of T2DM (12.28 > 10) (Supplementary Table 9, Model 1). Furthermore, no associations were observed between the T2DM polygenic score and cognitive status (Supplementary Table 9, Model 2), or history of T2DM and cognitive status (Supplementary Table 9, Model 3) in either logistic regression or Mendelian randomization analyses.

## Discussion

In a large sample of older Americans from the Health and Retirement Study (Wave, 2010), we examined the inferred causal relationship between T2DM and cognitive status using an inverse variance weighted Mendelian randomization framework. Using a cumulative genetic risk score for T2DM (polygenic score for T2DM) as a valid instrument, we observed a positive but non-causal association between history of T2DM and CIND in the European ancestry (*OR* = 1.30, 95% CI: 1.10, 1.52), after adjusting for age, sex, years of education, *APOE*-*ε4* allele status, and five ancestry-specific PCs. No association was found between history of T2DM and dementia.

We observed a significant association between history of T2DM and CIND in our European ancestry sample. Mechanistic studies suggest that T2DM may be linked with dementia through cardiovascular risk factors such as alterations in glucose, insulin, and amyloid metabolism (Biessels et al., 2006). For example, peripheral metabolic derangements from insulin resistance or T2DM may lead to desensitization of neuronal insulin receptors, which may in turn lead to decreased clearance of beta amyloid (Aβ) peptide and increased hyperphosphorylation of τ protein, which forms neurofibrillary tangles and damage the brain, but which of these are clinically relevant is unclear (Gasparini et al., 2001). The positive association we found between T2DM and CIND in the European ancestry sample was consistent with several previous cross-sectional studies of dementia (Haan et al., 2003; Valcour et al., 2005; Duron and Hanon, 2008; Gudala et al., 2013). A systematic review of 19 population-based longitudinal studies reported an estimated relative risk of 1.5–1.9 for all-cause dementia among people with T2DM (Peila et al., 2002; Xu et al., 2004; Østergaard et al., 2015). On the other hand, we observed a significant association between history of T2DM and dementia only in the older European ancestry sample (age between 65 and 90). One potential explanation is that people in the younger group are too young to develop dementia, which may bias the association toward null.

Additionally, we found no inferred causal association between history of T2DM and impaired cognition in our one-sample Mendelian randomization analyses, similar to Mendelian randomization analyses for Alzheimer’s disease phenotypes (Østergaard et al., 2015; Walter et al., 2016). Our research can be viewed in the context of several studies with similar research questions. All four of these studies found no evidence of a causal association between T2DM and Alzheimer’s disease (Østergaard et al., 2015; Walter et al., 2016; Benn et al., 2020; Thomassen et al., 2020). However, one of the studies observed a potential causal associations between insulin sensitivity and Alzheimer’s disease risk (Walter et al., 2016) and another observed a potential causal association between high plasma glucose and unspecified dementia (Benn et al., 2020). Evidence from these studies, as well as from this current study, suggest that though there does not seem to be an overall causal effect between T2DM and dementia, we may observe causal associations between mechanisms underlying T2DM (e.g., alterations in glucose, insulin resistance) and subtypes of dementia (e.g., unspecified dementia or Alzheimer’s disease). For two-sample Mendelian randomization, our study is likely to meet the two additional assumptions. Specifically, the two samples (T2DM and AD GWASs) represent the same underlying population and there were no overlapped participants between these two GWAS samples. We did not observe any significant causal association when using summary statistics from public GWASs, which further support our results of no causation from T2DM to dementia. However, according to our analysis, the associations between polygenic risk score for T2DM and potential confounders such as hypertension history, education, and BMI indicate a violation of the independence assumption of Mendelian randomization. Thus, the inferred causal results should be interpreted with caution.

Previous T2DM and dementia Mendelian randomization studies have used summary statistics for T2DM from the DIAbetes Genetics Replication And Meta-analysis consortium and for Alzheimer’s disease from the International Genomics of Alzheimer’s Project (IGAP)—two GWAS performed in the early 2010s (Morris et al., 2012; Lambert et al., 2013). A fourth study assessed summary statistics from the Meta-Analyses of Glucose and Insulin-related traits Consortium (Dupuis et al., 2010; Scott et al., 2012) and the IGAP Alzheimer’s disease summary statistics. These studies focused on genetic instruments for T2DM made from a minimal number of variants ranging from seven to several hundred. We have been able to use more recent summary statistics and a polygenic approach that incorporates hundreds of thousands of variants across the genome (Ware et al., 2017a; Xue et al., 2018; Kunkle et al., 2019). These prior Mendelian randomization studies were limited to white, European ancestry samples due in part to a lack of diversity in available genotype-phenotype data and the paucity of GWAS involving non-European populations. While we currently do not have GWAS in African ancestry populations that are comparable to the sizes we see in European ancestry populations, we were still able to provide preliminary associations between T2DM and cognitive status in a sample of almost 2,000 non-Hispanic Black Americans of African ancestry. Acknowledging the limitations of out-of-ancestry polygenic score associations, we show that our European GWAS based polygenic scores were weak instruments in our African sample.

Though we replicated our analyses in a younger age group (restricted to people aged between 50 and 80) to account for survival bias, our sample might still be biased toward healthier T2DM patients—individuals who survived or did not experience significant T2DM-related morbidity. Our estimate of the T2DM-dementia relationship is likely to be an underestimate. Our exposure (T2DM) measurement was self-reported, which might not be representative of actual T2DM cases. This self-report has previously been shown to have 87% sensitivity and 96% specificity for glycosylated hemoglobin (HbA1c)-defined diabetes among self-reported white HRS participants (White et al., 2014). Future research should combine with laboratory analysis to clinically confirm T2DM, either with serum glucose levels or with glycosylated hemoglobin levels (Medscape, 2020). Moreover, our study is unable to differentiate between different sub-types of dementia (e.g., Alzheimer’s disease), which may be more sensitive to glucose or insulin. We were, however, able to capture a broad definition of cognitive impairment through a clinically validated classification (Crimmins et al., 2011). Further, we selected a cross-sectional analysis plan to capture a large, diverse sample with genetic data and a wide distribution of cognition and history of T2DM to maximize power. A longitudinal framework would help account for the lifetime variation in the trait instrumented by the genes and the trajectory of dementia. Cognitive trajectories and incident dementia should be examined in future studies.

This study has several strengths. First, instead of instrumenting a single genetic variant, we used multiple variants (summarized in a polygenic score) to increase the explained fraction of genetic variation of T2DM, and thus, increase the power of testing our hypotheses and strength of the instrument. Second, though the classification of cognition into three categories does not allow us to detect specific dementia sub-types, we do identify an intermediate phenotype, CIND. As an advantage, we may identify a pre-clinical risk group which may be targeted for prevention and intervention. An additional advantage in our study is that we were able to include analyses on an a genetically homogeneous African ancestry sample, who are traditionally underrepresented in genetics research. Though this is one of the largest sample sizes for a US nationally representative African ancestry group, larger sample sizes in diverse ancestries are critically needed to translate precision medicine into practice (Landry et al., 2018; Sirugo et al., 2019). In addition, our polygenic scores were calculated using weights from European-only GWAS meta-analyses. The application of polygenic scores created from European summary statistics to non-European groups is documented (Martin et al., 2017; Ware et al., 2017b) to produce smaller and often non-significant effects. Likely as a direct result of this application, our polygenic scores were weak instruments in our African ancestry sample, and we were unable to perform Mendelian randomization analyses. This again highlights the necessity for large GWAS in non-European groups and reliable and valid methods to create polygenic scores in populations underrepresented in the genetic literature.

T2DM can be effectively prevented or improved by modifications to diet, physical exercise, and necessary medical treatments. Even though we found no causal relationship between T2DM and impaired cognition, the 20-30% prevalence of a history of T2DM in our samples is reflective of a high burden of disease in the population. Developing prevention and treatment methods early in the course of T2DM may lead to substantial reductions in the burden of T2DM in later years. And, with 17–36% of our sample classified as having some level of cognitive impairment, we continue to need investigations into causal effects of modifiable risk factors on dementia and cognitive impairment to reduce the increasing burden of this disease.

## Data Availability Statement

Publicly available datasets were analyzed in this study. This data can be found here: Health and Retirement Study health and covariate data are available here: https://hrs.isr.umich.edu/data-products. Health and Retirement Study genetic data are available here: dbGaP Study Accession: phs000428.v2.p2 (https://www.ncbi.nlm.nih.gov/projects/gap/cgi-bin/study.cgi?study_id=phs000428.v2.p2).

## Ethics Statement

The studies involving human participants were reviewed and approved by the University of Michigan Institutional Review Board (HUM00128220). The patients/participants provided their written informed consent to participate in this study.

## Author Contributions

EW prepared the dataset. CM and MF performed the analyses. CM wrote the first draft of the manuscript. EW, MF, and KB conducted the manuscript editing. EW and KB provided the project conceptualization and funding. All authors contributed to the article and approved the submitted version.

## Conflict of Interest

The authors declare that the research was conducted in the absence of any commercial or financial relationships that could be construed as a potential conflict of interest. The handling editor declared a past co-authorship with one of the authors EW.

## Footnotes

1 https://github.com/bakulskilab

## Abbreviations

AD: Alzheimer’s disease
APOE: Apolipoprotein E
*BMI*: *body mass index*
CI: confidence interval
GWAS: genome wide association study
HRS: Health and Retirement Study
OR: odds ratio
PGS: polygenic score.
