# Supplementary material for: Type 2 Diabetes and Cognitive Status in the Health and Retirement Study: A Mendelian Randomization Approach
Source: Front Genet. 2021 Mar 25;12:634767. doi: 10.3389/fgene.2021.634767 (PMC8044888; doi:10.3389/fgene.2021.634767)
Supplement: Supplementary file 1 [file Data_Sheet_1.docx]

**Type 2 diabetes and cognitive status in the Health and Retirement Study: A Mendelian randomization approach**

**Supplementary Material.**

This supplement contains 1 supplemental figure and 7 supplemental tables.

| **Supplementary Figure 1.** Sample selection steps in the Health and Retirement Study sample (Wave 2010) |  |
| --- | --- |
|  |  |
| 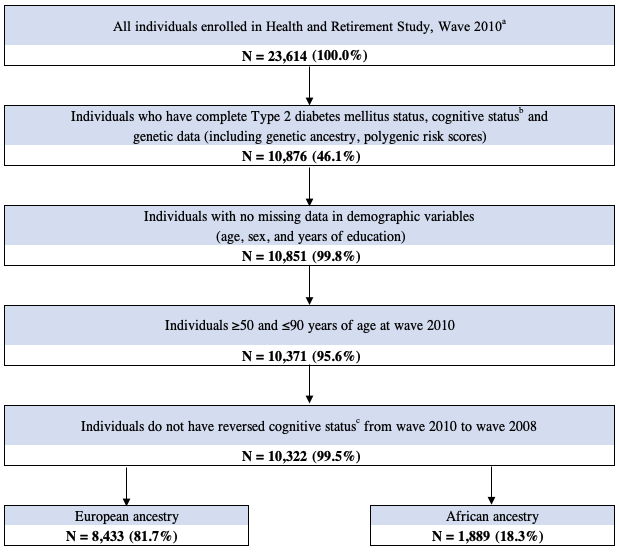 |  |
|  |  |
| **Notes:** [a] Samples were identified from the Health and Retirement Study database, which is a national longitudinal panel study of individuals aged over 50 in the United States.  [b] Cognitive status was categorized into three levels as normal, cognitive impairment non-dementia, and dementia based on results of a series of cognitive tests. The cut-point for categorization was established by Langa et al. 2009 (35). [c] Reversed cognition status refers to people classified as normal cognition in current wave (e.g. 2010) but dementia in the former wave (e.g. 2008). |  |
|  |  |
|  |  |
|  |  |
|  |  |
|  |  |

| **Supplementary Table 1.** Univariate characteristics of study participants in the Health and Retirement Study sample, Wave 2010: included (n = 10322) vs. excluded sample (n = 13292)^a^ | | | | |  |
| --- | --- | --- | --- | --- | --- |
|  |  |  |  |  |  |
|  | **Overall** | **Included** | **Excluded** | **P-value^b^** |  |
|  | **n = 23614** | **n = 10322** | **n = 13292** |  |  |
| History of Type 2 diabetes mellitus (Yes) | 4803 (21.8%) | 2161 (20.9%) | 2642 (22.6%) | 0.002* |  |
| Cognitive Status at Wave 2010 |  |  |  | <0.001* |  |
| Normal | 16704 (75.8%) | 8186 (79.3%) | 8518 (72.8%) |  |  |
| Cognitive Impairment Non-Dementia | 3859 (17.5%) | 1642 (15.9%) | 2217 (18.9%) |  |  |
| Dementia | 1465 (6.65%) | 494 (4.79%) | 971 (8.29%) |  |  |
| *APOE-ε4* allele carrier (Yes) | 3984 (27.0%) | 2953 (28.6%) | 1031 (23.2%) | <0.001* |  |
| Sex (Female) | 13704 (58.0%) | 6034 (58.5%) | 7670 (57.7%) | 0.25 |  |
| Genetic ancestry (European) | 11462 (79.5%) | 8433 (81.7%) | 3029 (73.9%) | <0.001* |  |
| Stroke history (Yes) | 1652 (7.50%) | 801 (7.76%) | 851 (7.27%) | 0.18 |  |
| Hypertension history (Yes) | 12602 (57.3%) | 6259 (60.7%) | 6343 (54.3%) | <0.001* |  |
| Smoking status |  |  |  | <0.001* |  |
| Never | 9522 (43.5%) | 4392 (42.8%) | 5130 (44.1%) |  |  |
| Former | 8956 (40.9%) | 4512 (44.0%) | 4444 (38.2%) |  |  |
| Current | 3423 (15.6%) | 1359 (13.2%) | 2064 (17.7%) |  |  |
| Drink status (Ever drinker) | 12296 (55.8%) | 5638 (54.6%) | 6658 (56.9%) | 0.001* |  |
| Proxy status (Self-respondent) | 20650 (93.7%) | 10051 (97.4%) | 10599 (90.5%) | <0.001* |  |
| Age at 2010 (yrs) | 66.9 (12.5) | 68.8 (10.2) | 65.4 (13.9) | <0.001* |  |
| Years of education | 12.5 (3.27) | 13.1 (2.60) | 12.1 (3.65) | <0.001* |  |
| Body Mass Index (kg/m^2^) | 28.5 (6.20) | 28.4 (6.11) | 28.6 (6.27) | 0.01* |  |
| *Abbreviations: APOE, Apolipoprotein E.* | | | | |  |
| **Notes:** [a] All the statistics including count, frequency, mean, standard deviation, and *P* value were calculated based on non-missing data for each variable. [b] The *P* value was calculated from chi-square test or analysis of variance for categorical or continuous variables as appropriate, interpreted as differences between groups. The asterisk indicates statistical significance at 0.05 level. | | | | |  |
|  |  |  |  |  |  |
|  |  |  |  |  |  |
|  |  |  |  |  |  |

| **Supplementary Table 2.** Characteristics of eligible participants (n = 10322) stratified by ancestry, Health and Retirement Study, Wave 2010^a^ | | | | |  |
| --- | --- | --- | --- | --- | --- |
|  | **Overall** | **European** | **African** | ***P*-value^b^** |  |
|  | **n = 10322** | **n = 8433** | **n = 1889** |  |  |
| History of Type 2 diabetes mellitus (Yes) | 2161 (20.9%) | 1601 (19.0%) | 560 (29.6%) | <0.001* |  |
| Cognitive status at Wave 2010 |  |  |  | <0.001* |  |
| Normal | 8186 (79.3%) | 6995 (82.9%) | 1191 (63.0%) |  |  |
| Cognitive Impairment-Non Dementia | 1642 (15.9%) | 1119 (13.3%) | 523 (27.7%) |  |  |
| Dementia | 494 (4.79%) | 319 (3.78%) | 175 (9.26%) |  |  |
| *APOE-ε4* allele carrier (Yes) | 2953 (28.6%) | 2233 (26.5%) | 720 (38.1%) | <0.001* |  |
| Sex (Female) | 6034 (58.5%) | 4860 (57.6%) | 1174 (62.1%) | <0.001* |  |
| Stroke history (Yes) | 801 (7.76%) | 611 (7.25%) | 190 (10.1%) | <0.001* |  |
| Hypertension history (Yes) | 6259 (60.7%) | 4874 (57.9%) | 1385 (73.4%) | <0.001* |  |
| Smoking status |  |  |  | <0.001* |  |
| Never | 4392 (42.8%) | 3647 (43.5%) | 745 (39.6%) | <0.001*  <0.001* |  |
| Former | 4512 (44.0%) | 3768 (44.9%) | 744 (39.6%) |  |  |
| Current | 1359 (13.2%) | 969 (11.6%) | 390 (20.8%) |  |  |
| Drink status (Ever drinker) | 5638 (54.6%) | 4782 (56.7%) | 856 (45.3%) |  |  |
| Proxy status (Self-respondent) | 10051 (97.4%) | 8200 (97.2%) | 1851 (98.0%) | 0.08 |  |
| Age at 2010 (yrs) | 68.8 (10.2) | 69.6 (10.1) | 65.2 (9.81) | <0.001* |  |
| Years of education | 13.1 (2.60) | 13.3 (2.49) | 12.2 (2.88) | <0.001* |  |
| Body Mass Index (kg/m^2^) | 28.4 (6.11) | 28.0 (5.79) | 30.2 (7.11) | <0.001* |  |
| *Abbreviations: APOE, Apolipoprotein E; SD: standard deviation.* |  |  |  |  |  |
| **Notes:** [a] All the statistics including count, frequency, mean, standard deviation, and *P* value were calculated based on non-missing data for each variable. [b] The *P* value was calculated from chi-square test or analysis of variance for categorical or continuous variables as appropriate, interpreted as differences between groups. The asterisk indicates statistical significance at 0.05 level.  [c] The Type 2 diabetes mellitus polygenic score was created using weights from a genome-wide association study meta-analysis from the DIAbetes Genetics Replication and Meta-analysis Consortium (10) using a *P*-value threshold of 1.  [d] The Alzheimer’s disease polygenic score was created using weights from a genome-wide association study meta-analysis from a 2019 GWAS by Kunkle et al. (22) using a *P*-value threshold of 0.01 and removing the *APOE* region (chr19: 45,384,477 to 45,432,606, build 37/hg 19) (21). | | | | |  |
|  | | | | |  |
|  |  |  |  |  |  |
|  |  |  |  |  |  |
|  |  |  |  |  |  |

| **Supplementary Table 3.** Bivariate characteristics stratified by cognitive status or Type 2 diabetes mellitus (T2DM) status, Health and Retirement Study, Wave 2010, African ancestry sample (n = 1889)^a^ | | | | | | | | | | |  |
| --- | --- | --- | --- | --- | --- | --- | --- | --- | --- | --- | --- |
|  |  |  |  |  |  |  |  |  |  |  |  |
|  | **Cognitive Status** | | | | |  | **Type 2 diabetes mellitus status** | | | |  |
|  | **Overall** | **Normal** | **CIND** | **Dementia** | **P-value^b^** |  | **Overall** | **Yes** | **No** | **P-value^b^** |  |
|  | **n = 1889** | **n = 1191** | **n = 523** | **n = 175** |  |  | **n = 1889** | **n = 560** | **n = 1329** |  |  |
| **Categorical Variables [Count (Frequency)]** | |  |  |  |  |  |  |  |  |  |  |
| T2DM status (Yes) | 560 (29.6%) | 322 (27.0%) | 182 (34.8%) | 56 (32.0%) | 0.004* |  | - | | | |  |
| Sex (Female) | 1174 (62.1%) | 765 (64.2%) | 310 (59.3%) | 99 (56.6%) | 0.04* |  | 1174 (62.1%) | 350 (62.5%) | 824 (62.0%) | 0.88 |  |
| Stroke history (Yes) | 190 (10.1%) | 87 (7.30%) | 57 (10.9%) | 46 (26.3%) | <0.001* |  | 190 (10.1%) | 84 (15.0%) | 106 (7.98%) | <0.001* |  |
| Hypertension history (Yes) | 1385 (73.4%) | 840 (70.6%) | 401 (76.8%) | 144 (82.3%) | 0.001* |  | 1385 (73.4%) | 494 (88.2%) | 891 (67.1%) | <0.001* |  |
| Smoking status |  |  |  |  | 0.31 |  |  |  |  | <0.001* |  |
| Never | 745 (39.6%) | 481 (40.5%) | 195 (37.6%) | 69 (39.9%) |  |  | 745 (39.6%) | 211 (37.9%) | 534 (40.4%) |  |  |
| Former | 744 (39.6%) | 452 (38.1%) | 216 (41.6%) | 76 (43.9%) |  |  | 744 (39.6%) | 257 (46.2%) | 487 (36.8%) |  |  |
| Current | 390 (20.8%) | 254 (21.4%) | 108 (20.8%) | 28 (16.2%) |  |  | 390 (20.8%) | 88 (15.8%) | 302 (22.8%) |  |  |
| Drink status (Ever drinker) | 856 (45.3%) | 603 (50.6%) | 212 (40.6%) | 41 (23.4%) | <0.001* |  | 856 (45.3%) | 202 (36.1%) | 654 (49.2%) | <0.001* |  |
| *APOE-ε4* allele carrier (Yes) | 720 (38.1%) | 443 (37.2%) | 192 (36.7%) | 85 (48.6%) | 0.011 |  | 720 (38.1%) | 221 (39.5%) | 499 (37.5%) | 0.46 |  |
| **Continuous Variables [Mean (SD)]** |  |  |  |  |  |  |  |  |  |  |  |
| T2DM polygenic score | 0.01 (0.99) | -0.02 (1.01) | 0.01 (0.96) | 0.14 (0.98) | 0.14 |  | 0.01 (0.99) | 0.11 (0.97) | -0.04 (1.00) | 0.003* |  |
| Alzheimer's disease polygenic score | 0.00 (0.98) | -0.04 (1.01) | -0.01 (0.94) | 0.26 (0.88) | 0.001* |  | 0.00 (0.98) | 0.02 (1.00) | -0.02 (0.98) | 0.40 |  |
| Age at 2010 (yrs) | 65.2 (9.81) | 62.8 (8.71) | 67.3 (9.88) | 75.1 (9.17) | <0.001* |  | 65.2 (9.81) | 67.0 (9.08) | 64.4 (10.0) | <0.001* |  |
| Years of education | 12.2 (2.88) | 13.0 (2.38) | 11.3 (2.68) | 9.18 (3.60) | <0.001* |  | 12.2 (2.88) | 11.8 (3.01) | 12.4 (2.80) | <0.001* |  |
| Body Mass Index (kg/m^2^) | 30.2 (7.11) | 30.7 (7.00) | 30.0 (7.07) | 27.4 (7.33) | <0.001* |  | 30.2 (7.11) | 32.1 (7.60) | 29.4 (6.73) | <0.001* |  |
| *Abbreviations: APOE, Apolipoprotein E; CIND, cognitive impairment non-dementia; SD: standard deviation; T2DM: Type 2 diabetes mellitus.* | | | | | | | | | | |  |
| **Notes:** [a] All the statistics including count, frequency, mean, standard deviation, and *P* value were calculated based on non-missing data for each variable. [b] The overall *P* value was calculated from chi-square test or analysis of variance for categorical or continuous variables as appropriate, interpreted as differences between groups. | | | | | | | | | | |  |
|  |  |  |  |  |  |  |  |  |  |  |  |
|  |  |  |  |  |  |  |  |  |  |  |  |

| **Supplementary Table 4.** Associations between polygenic score for Type 2 diabetes mellitus and cognitive status in the subsample of people with no diabetes history, Health and Retirement Study, Wave 2010, European ancestry sample (n = 6711)^a^ | | | | | | | |  |
| --- | --- | --- | --- | --- | --- | --- | --- | --- |
|  |  |  |  |  |  |  |  |  |
|  |  |  |  |  |  |  |  |  |
|  | **CIND vs. Normal cognition sample**  **n = 6478** | | |  | **Dementia vs. Normal cognition sample**  **n = 5886** | | |  |
|  |  | **OR** | **(95 CI%)** |  |  | **OR** | **(95 CI%)** |  |
| **Total effect of T2DM polygenic score^b^** | | |  |  |  |  |  |  |
| Crude |  | 0.99 | (0.92, 1.06) |  |  | 0.97 | (0.85, 1.10) |  |
| Adjusted (primary)^c^ |  | 1.04 | (0.95, 1.13) |  |  | 1.06 | (0.90, 1.24) |  |
| Adjusted (health status)^d^ |  | 1.03 | (0.94, 1.12) |  |  | 1.05 | (0.89, 1.24) |  |
| Adjusted (AD genetics)^e^ |  | 1.03 | (0.94, 1.12) |  |  | 1.04 | (0.88, 1.23) |  |
| *Abbreviations: AD, Alzheimer's disease; APOE, Apolipoprotein E; BMI, body mass index; CI, confidence interval; CIND, cognitive impairment-non dementia; OR, odds ratio.* | | | | | | | |  |
|  |  |  |  |  |  |  |  |  |
| **Notes:** [a] All the values were based on results from multivariable logistic regression analyses in each sample, in which "normal cognitive status" was used as the reference group.  [b] The Type 2 diabetes mellitus polygenic score was created using weights from a genome-wide association study meta-analysis from the DIAbetes Genetics Replication and Meta-analysis Consortium (10) using a P-value threshold of 1. [c] Adjusted for age, sex, years of education, *APOE-ε4* allele status, and five ancestry-specific principal component sets. [d] Adjusted for smoking status, ever drink alcohol, history of stroke, hypertension, and BMI in addition to variables in [c]. [e] Adjusted for Alzheimer's disease polygenic score in addition to variables in [d]. The Alzheimer’s disease polygenic score was created using weights from a genome-wide association study meta-analysis from a 2019 GWAS by Kunkle et al. (22) using a P-value threshold of 0.01 and removing the APOE region (chr19: 45,384,477 to 45,432,606, build 37/hg 19) (21). | | | | | | | |  |
|  |  |  |  |  |  |  |  |  |
|  |  |  |  |  |  |  |  |  |
|  |  |  |  |  |  |  |  |  |
|  |  |  |  |  |  |  |  |  |

| **Supplementary Table 5.** Results from Mendelian randomization of Type 2 diabetes mellitus status on cognitive status using different *P* value thresholds for polygenic scores of Type 2 diabetes mellitus, Health and Retirement Study, Wave 2010, European ancestry sample (n = 8433)^a^ | | | | | | | | | | | | | |  |
| --- | --- | --- | --- | --- | --- | --- | --- | --- | --- | --- | --- | --- | --- | --- |
|  |  |  |  |  |  |  |  |  |  |  |  |  |  |  |
|  | **Results from multivariable logistic regression** | | | **Mendelian randomization results (Wald-type/ratio) on different  *P* value thresholds of T2DM polygenic score^b^** | | | | | | | | | |  |
|  |  |  |  | ***P* < 0.3** | | ***P* < 0.1** | | ***P* < 0.05** | | ***P* < 0.01** | | ***P* < 0.001** | |  |
|  |  | **OR** | **(95 CI%)** | **OR** | **(95 CI%)** | **OR** | **(95 CI%)** | **OR** | **(95 CI%)** | **OR** | **(95 CI%)** | **OR** | **(95 CI%)** |  |
| ***CIND vs. Normal cognition (n=7979)*** |  |  |  |  |  |  |  |  |  |  |  |  |  |  |
| Crude |  | 1.56 | (1.34, 1.81) | 1.00 | (0.86, 1.16) | 0.99 | (0.86, 1.15) | 0.99 | (0.86, 1.15) | 0.97 | (0.84, 1.13) | 0.98 | (0.84, 1.15) |  |
| Adjusted (primary)^c^ |  | 1.30 | (1.10, 1.52) | 1.04 | (0.90, 1.21) | 1.04 | (0.90, 1.20) | 1.04 | (0.90, 1.20) | 1.03 | (0.89, 1.18) | 1.03 | (0.89, 1.20) |  |
| Adjusted (health status)^d^ |  | 1.30 | (1.09, 1.54) | 1.01 | (0.88, 1.18) | 1.01 | (0.87, 1.17) | 1.01 | (0.88, 1.17) | 1.00 | (0.87, 1.16) | 1.01 | (0.87, 1.18) |  |
| Adjusted (AD genetics)^e^ |  | 1.30 | (1.09, 1.54) | 1.01 | (0.88, 1.18) | 1.01 | (0.88, 1.17) | 1.01 | (0.88, 1.17) | 1.00 | (0.87, 1.16) | 1.01 | (0.87, 1.18) |  |
| ***Dementia vs. Normal cognition (n=7186)*** |  |  |  |  |  |  |  |  |  |  |  |  |  |  |
| Crude |  | 1.53 | (1.17, 1.98) | 1.06 | (0.82, 1.36) | 1.08 | (0.84, 1.39) | 1.11 | (0.86, 1.43) | 1.11 | (0.85, 1.45) | 1.19 | (0.89, 1.59) |  |
| Adjusted (primary) |  | 1.28 | (0.95, 1.72) | 1.21 | (0.94, 1.55) | 1.23 | (0.97, 1.58) | 1.26 | (0.99, 1.62) | 1.21 | (0.94, 1.56) | 1.25 | (0.94, 1.64) |  |
| Adjusted (health status) |  | 1.36 | (0.98, 1.87) | 1.18 | (0.91, 1.53) | 1.21 | (0.94, 1.57) | 1.24 | (0.95, 1.60) | 1.20 | (0.92, 1.56) | 1.21 | (0.90, 1.61) |  |
| Adjusted (AD genetics) |  | 1.34 | (0.97, 1.85) | 1.18 | (0.91, 1.53) | 1.21 | (0.94, 1.57) | 1.23 | (0.95, 1.60) | 1.20 | (0.92, 1.57) | 1.21 | (0.91, 1.62) |  |
| *Abbreviations: AD, Alzheimer's disease; APOE, Apolipoprotein E; BMI, body mass index; CI: confidence interval; CIND: cognitive impairment-non dementia; OR: odds ratio; T2DM: Type 2 diabetes mellitus.* | | | | | | | | | | | | | |  |
| **Notes:** [a] All values were based on results from multivariable logistic regression or Mendelian randomization analyses in each sample, in which "normal cognitive status" and "no diabetes history" were used as reference groups.  [b] The Type 2 diabetes mellitus polygenic score was created using weights from a genome-wide association study meta-analysis from the DIAbetes Genetics Replication and Meta-analysis Consortium (10) using a P-value threshold of 1.  [c] Adjusted for age, sex, years of education, *APOE-ε4* allele status, and five ancestry-specific principal component sets. [d] Adjusted for smoking status, ever drink alcohol, history of stroke, hypertension, and BMI in addition to variables in [c]. [e] Adjusted for Alzheimer's disease polygenic score in addition to variables in [d]. The Alzheimer’s disease polygenic score was created using weights from a genome-wide association study meta-analysis from a 2019 GWAS by Kunkle et al. (22) using a P-value threshold of 0.01 and removing the APOE region (chr19: 45,384,477 to 45,432,606, build 37/hg 19) (21). | | | | | | | | | | | | | |  |
|  |  |  |  |  |  |  |  |  |  |  |  |  |  |  |
|  |  |  |  |  |  |  |  |  |  |  |  |  |  |  |
|  |  |  |  |  |  |  |  |  |  |  |  |  |  |  |

| **Supplementary Table 6.** Associations between polygenic score for Type 2 diabetes mellitus, history of Type 2 diabetes mellitus, and cognitive status, Health and Retirement Study, Wave 2010, younger European ancestry sample (n = 6952)^a^ | | | | | | | | | | | |  |
| --- | --- | --- | --- | --- | --- | --- | --- | --- | --- | --- | --- | --- |
|  |  |  |  |  |  |  |  |  |  |  |  |  |
| **Model 1: History of T2DM ~ T2DM polygenic score^b^** | | | | | | | | | | | |  |
|  |  | **Overall sample  (n = 6952)** | | | **CIND & Normal cognition sample (n = 6818)** | | | | **Dementia & Normal cognition sample (n = 6231)** | | |  |
|  |  | **OR (95 CI%)** | | | **OR (95 CI%)** | | | | **OR (95 CI%)** | | |  |
| Crude | | 1.53 (1.43, 1.63) | | | 1.53 (1.43, 1.63) | | | |  | 1.53 (1.44, 1.63) | |  |
| Adjusted (primary)^c^ | | 1.65 (1.54, 1.77) | | | 1.67 (1.55, 1.80) | | | |  | 1.65 (1.54, 1.77) | |  |
| Improvement ꭓ^2 d^ | | 200.8 | | | 188.8 | | | |  | 208.3 | |  |
| **Model 2: Cognitive Status ~ T2DM polygenic score** | | | | | | | | | | | |  |
|  |  |  | **CIND & Normal cognition sample**  **(n = 6818)** | | | | | **Dementia & Normal cognition sample**  **(n = 6231)** | | | |  |
|  |  |  |  | **OR** | **(95 CI%)** |  |  |  | **OR** | **(95 CI%)** |  |  |
| **Total effect of T2DM polygenic score** | | |  | | | | | | | | |  |
| Crude | | |  | 1.04 | (0.96, 1.12) |  |  |  | 1.17 | (0.99, 1.39) |  |  |
| Adjusted (primary)^c^ | | |  | 1.02 | (0.93, 1.11) |  |  |  | 1.16 | (0.95, 1.40) |  |  |
| Adjusted (health status)^e^ | | |  | 0.99 | (0.91, 1.09) |  |  |  | 1.13 | (0.92, 1.38) |  |  |
| Adjusted (AD genetics)^f^ | | |  | 0.99 | (0.91, 1.09) |  |  |  | 1.13 | (0.92, 1.38) |  |  |
| **Direct effect of T2DM polygenic score (adjusting for history of T2DM)** | | | | | | | |  |  |  |  |  |
| Crude | | |  | 1.00 | (0.93, 1.09) |  |  |  | 1.14 | (0.96, 1.35) |  |  |
| Adjusted (primary) | | |  | 0.99 | (0.91, 1.09) |  |  |  | 1.14 | (0.94, 1.39) |  |  |
| Adjusted (health status) | | |  | 0.98 | (0.89, 1.07) |  |  |  | 1.11 | (0.91, 1.36) |  |  |
| Adjusted (AD genetics) | | |  | 0.98 | (0.89, 1.07) |  |  |  | 1.11 | (0.91, 1.36) |  |  |
| **Model 3: Cognitive Status ~ History of T2DM** | | | | | | | | | | | |  |
|  | **CIND & Normal cognition sample (n = 6818)** | | | | |  | **Dementia & Normal cognition sample (n = 6231)** | | | | |  |
|  | **Logistic Regression** | | **Wald-type/ratio** | | ***P* for heterogeneity^g^** |  | **Logistic Regression** | | **Wald-type/ratio** | | ***P* for heterogeneity** |  |
|  | **OR** | **(95 CI%)** | **OR** | **(95 CI%)** |  |  | **OR** | **(95 CI%)** | **OR** | **(95 CI%)** |  |  |
| Crude | 1.63 | (1.36, 1.95) | 1.10 | (0.91, 1.32) | 0.003* |  | 1.58 | (1.05, 2.32) | 1.45 | (0.97, 2.17) | 0.76 |  |
| Adjusted (primary) | 1.31 | (1.08, 1.58) | 1.03 | (0.86, 1.23) | 0.07 |  | 1.27 | (0.83, 1.90) | 1.33 | (0.91, 1.93) | 0.87 |  |
| Adjusted (health status) | 1.26 | (1.03, 1.54) | 0.99 | (0.82, 1.18) | 0.08 |  | 1.33 | (0.84, 2.07) | 1.26 | (0.86, 1.86) | 0.86 |  |
| Adjusted (AD genetics) | 1.26 | (1.03, 1.54) | 0.99 | (0.82, 1.18) | 0.08 |  | 1.31 | (0.83, 2.05) | 1.26 | (0.85, 1.86) | 0.90 |  |
| *Abbreviations: AD, Alzheimer's disease; APOE, Apolipoprotein E; BMI, body mass index; CI: confidence interval; CIND: cognitive impairment-non dementia; OR: odds ratio; T2DM: Type 2 diabetes mellitus.* | | | | | | | | | | | |  |
| **Notes:** [a] All the values were based on results from multivariable logistic regression or Mendelian randomization analyses in each sample, in which "normal cognitive status" and "no diabetes history" were used as reference groups.  [b] The Type 2 diabetes mellitus polygenic score was created using weights from a genome-wide association study meta-analysis from the DIAbetes Genetics Replication and Meta-analysis Consortium (10) using a *P*-value threshold of 1. [c] Adjusted for age, sex, years of education, APOE-ε4 allele status, and five ancestry-specific principal component sets. [d] Calculated by 2*(log likelihood of full model - log likelihood of reduced model). By convention, statistics larger than 10 indicate a valid instrument. [e] Adjusted for smoking status, ever drink alcohol, history of stroke, hypertension, and BMI in addition to variables in [c]. [f] Adjusted for Alzheimer's disease polygenic score in addition to variables in [e]. The Alzheimer’s disease polygenic score was created using weights from a genome-wide association study meta-analysis from a 2019 GWAS by Kunkle et al. (22) using a P-value threshold of 0.01 and removing the APOE region (chr19: 45,384,477 to 45,432,606, build 37/hg 19) (21). [g] *P* value represents the statistical significance of the test of heterogeneity between logistic regression and Mendelian randomization. | | | | | | | | | | | |  |
|  |  |  |  |  |  |  |  |  |  |  |  |  |
|  |  |  |  |  |  |  |  |  |  |  |  |  |
|  |  |  |  |  |  |  |  |  |  |  |  |  |
|  |  |  |  |  |  |  |  |  |  |  |  |  |
|  |  |  |  |  |  |  |  |  |  |  |  |  |
|  |  |  |  |  |  |  |  |  |  |  |  |  |

| **Supplementary Table 7.** Associations between polygenic score for Type 2 diabetes mellitus, history of Type 2 diabetes mellitus, and cognitive status, Health and Retirement Study, Wave 2010, older European ancestry sample (n = 5479)^a^ | | | | | | | | | | | |  |
| --- | --- | --- | --- | --- | --- | --- | --- | --- | --- | --- | --- | --- |
|  |  |  |  |  |  |  |  |  |  |  |  |  |
| **Model 1: History of T2DM ~ T2DM polygenic score^b^** | | | | | | | | | | | |  |
|  |  | **Overall sample  (n = 5479)** | | | **CIND & Normal cognition sample (n = 5177)** | | | | **Dementia & Normal cognition sample (n = 4541)** | | |  |
|  |  | **OR (95 CI%)** | | | **OR (95 CI%)** | | | | **OR (95 CI%)** | | |  |
| Crude | | 1.45 (1.36, 1.56) | | | 1.48 (1.37, 1.59) | | | |  | 1.47 (1.38, 1.58) | |  |
| Adjusted (primary)^c^ | | 1.55 (1.44, 1.68) | | | 1.60 (1.17, 1.35) | | | |  | 1.58 (1.46, 1.70) | |  |
| Improvement ꭓ^2 d^ | | 127.6 | | | 126.5 | | | |  | 146.1 | |  |
| **Model 2: Cognitive Status ~ T2DM polygenic score** | | | | | | | | | | | |  |
|  |  |  | **CIND & Normal cognition sample**  **(n = 5177)** | | | | | **Dementia & Normal cognition sample**  **(n = 4541)** | | | |  |
|  |  |  |  | **OR** | **(95 CI%)** |  |  |  | **OR** | **(95 CI%)** |  |  |
| **Total effect of T2DM polygenic score** | | |  | | | | | | | | |  |
| Crude | | |  | 1.00 | (0.93, 1.07) |  |  |  | 1.04 | (0.93, 1.17) |  |  |
| Adjusted (primary)^c^ | | |  | 1.02 | (0.94, 1.11) |  |  |  | 1.10 | (0.96, 1.26) |  |  |
| Adjusted (health status)^e^ | | |  | 1.00 | (0.92, 1.09) |  |  |  | 1.08 | (0.94, 1.25) |  |  |
| Adjusted (AD genetics)^f^ | | |  | 1.00 | (0.92, 1.09) |  |  |  | 1.08 | (0.94, 1.25) |  |  |
| **Direct effect of T2DM polygenic score (adjusting for history of T2DM)** | | | | | | | |  |  |  |  |  |
| Crude | | |  | 0.98 | (0.91, 1.05) |  |  |  | 1.02 | (0.91, 1.15) |  |  |
| Adjusted (primary) | | |  | 1.00 | (0.92, 1.09) |  |  |  | 1.08 | (0.94, 1.24) |  |  |
| Adjusted (health status) | | |  | 0.98 | (0.90, 1.07) |  |  |  | 1.06 | (0.91, 1.22) |  |  |
| Adjusted (AD genetics) | | |  | 0.98 | (0.90, 1.07) |  |  |  | 1.06 | (0.91, 1.22) |  |  |
| **Model 3: Cognitive Status ~ History of T2DM** | | | | | | | | | | | |  |
|  | **CIND & Normal cognition sample (n = 5177)** | | | | |  | **Dementia & Normal cognition sample (n = 4541)** | | | | |  |
|  | **Logistic Regression** | | **Wald-type/ratio** | | ***P* for heterogeneity^g^** |  | **Logistic Regression** | | **Wald-type/ratio** | | ***P* for heterogeneity** |  |
|  | **OR** | **(95 CI%)** | **OR** | **(95 CI%)** |  |  | **OR** | **(95 CI%)** | **OR** | **(95 CI%)** |  |  |
| Crude | 1.37 | (1.16, 1.62) | 0.99 | (0.82, 1.20) | 0.01* |  | 1.37 | (1.04, 1.79) | 1.12 | (0.83, 1.50) | 0.33 |  |
| Adjusted (primary) | 1.27 | (1.06, 1.52) | 1.04 | (0.86, 1.26) | 0.14 |  | 1.32 | (0.97, 1.76) | 1.23 | (0.92, 1.65) | 0.74 |  |
| Adjusted (health status) | 1.27 | (1.05, 1.53) | 1.00 | (0.82, 1.21) | 0.08 |  | 1.41 | (1.01, 1.95) | 1.19 | (0.87, 1.61) | 0.46 |  |
| Adjusted (AD genetics) | 1.27 | (1.05, 1.53) | 1.00 | (0.82, 1.21) | 0.08 |  | 1.39 | (1.00, 1.92) | 1.19 | (0.87, 1.61) | 0.50 |  |
| *Abbreviations: AD, Alzheimer's disease; APOE, Apolipoprotein E; BMI, body mass index; CI: confidence interval; CIND: cognitive impairment-non dementia; OR: odds ratio; T2DM: Type 2 diabetes mellitus.* | | | | | | | | | | | |  |
| **Notes:** [a] All the values were based on results from multivariable logistic regression or Mendelian randomization analyses in each sample, in which "normal cognitive status" and "no diabetes history" were used as reference groups.  [b] The Type 2 diabetes mellitus polygenic score was created using weights from a genome-wide association study meta-analysis from the DIAbetes Genetics Replication and Meta-analysis Consortium (10) using a *P*-value threshold of 1. [c] Adjusted for age, sex, years of education, APOE-ε4 allele status, and five ancestry-specific principal component sets. [d] Calculated by 2*(log likelihood of full model - log likelihood of reduced model). By convention, statistics larger than 10 indicate a valid instrument. [e] Adjusted for smoking status, ever drink alcohol, history of stroke, hypertension, and BMI in addition to variables in [c]. [f] Adjusted for Alzheimer's disease polygenic score in addition to variables in [e]. The Alzheimer’s disease polygenic score was created using weights from a genome-wide association study meta-analysis from a 2019 GWAS by Kunkle et al. (22) using a P-value threshold of 0.01 and removing the APOE region (chr19: 45,384,477 to 45,432,606, build 37/hg 19) (21). [g] *P* value represents the statistical significance of the test of heterogeneity between logistic regression and Mendelian randomization. | | | | | | | | | | | |  |
|  |  |  |  |  |  |  |  |  |  |  |  |  |
|  |  |  |  |  |  |  |  |  |  |  |  |  |
|  |  |  |  |  |  |  |  |  |  |  |  |  |
|  |  |  |  |  |  |  |  |  |  |  |  |  |
|  |  |  |  |  |  |  |  |  |  |  |  |  |
|  |  |  |  |  |  |  |  |  |  |  |  |  |

| **Supplementary Table 8.** Associations between cognitive status on Type 2 diabetes mellitus status, Health and Retirement Study, Wave 2010, European ancestry sample (n = 8433)^a^ | | | | | | | | | | |  |
| --- | --- | --- | --- | --- | --- | --- | --- | --- | --- | --- | --- |
|  |  |  |  |  |  |  |  |  |  |  |  |
| **Model 1: Cognitive status ~ AD PGS^b^** | | | | | | | | | | |  |
|  |  | **CIND & Normal cognition sample**  **(n = 7979)** | | |  |  | **Dementia & Normal cognition sample (n = 6231)** | | | |  |
|  |  | **OR** | **(95 CI%)** | |  |  | **OR** | | **(95 CI%)** | |  |
| Crude | | 1.03 | (0.97, 1.10) | |  |  | 1.17 | | (1.04, 1.31) | |  |
| Adjusted (primary)^c^ | | 1.01 | (0.94, 1.08) | |  |  | 1.19 | | (1.05, 1.35) | |  |
| Improvement ꭓ^2 d^ | | 2050.8 | | |  |  | 4819.7 | | | |  |
| **Model 2: History of T2DM ~ Cognitive status** | | | | | | | | | | |  |
|  | **CIND & Normal cognition sample** | | | |  | **Dementia & Normal cognition sample** | | | | |  |
|  | **Logistic Regression** | | **Wald-type/ratio** | |  | **Logistic Regression** | | | **Wald-type/ratio** | |  |
|  | **OR** | **(95 CI%)** | **OR** | **(95 CI%)** |  | **OR** | **(95 CI%)** | **OR** | | **(95 CI%)** |  |
| Crude | 1.56 | (1.34, 1.81) | 0.66 | (0.09, 4.61) |  | 1.53 | (1.17, 1.98) | 0.93 | | (0.63, 1.37) |  |
| Adjusted (primary)^c^ | 1.23 | (1.05, 1.45) | 0.01 | (0.00, 11.53) |  | 1.10 | (0.82, 1.45) | 0.85 | | (0.59, 1.21) |  |
| Adjusted (health status)^e^ | 1.24 | (1.05, 1.47) | 0.04 | (0.00, 54.25) |  | 1.14 | (0.84, 1.53) | 0.91 | | (0.63, 1.33) |  |
| Adjusted (T2DM genetics)^f^ | 1.25 | (1.05, 1.49) | 0.08 | (0.00, 110.76) |  | 1.12 | (0.82, 1.52) | 0.94 | | (0.64, 1.38) |  |
| *Abbreviations: CI, confidence interval; CIND, cognitive impairment-non dementia; OR: Odds Ratio; PC: principal components; T2DM: Type 2 diabetes.* | | | | | | | | | | |  |
| **Notes:** [a] All the values were based on results from multivariable logistic regression or Mendelian randomization analyses in each sample, in which "normal cognitive status" and "no diabetes history" were used as reference groups.  [b] The Alzheimer’s disease polygenic score was created using weights from a genome-wide association study meta-analysis from a 2019 GWAS by Kunkle et al. (22) using a P-value threshold of 0.01 and removing the APOE region (chr19: 45,384,477 to 45,432,606, build 37/hg 19) (21).  [c] Adjusted for age, sex, years of education, *APOE-ε4* allele status, and five ancestry-specific principal component sets. [d] Calculated by 2*(log likelihood of full model - log likelihood of reduced model). By convention, statistics larger than 10 indicate a valid instrument. [e] Adjusted for smoking status, ever drink alcohol, history of stroke, hypertension, and BMI in addition to variables in [c]. [f] Adjusted for T2DM polygenic score in addition to variables in [e]. The Type 2 diabetes mellitus polygenic score was created using weights from a genome-wide association study meta-analysis from the DIAbetes Genetics Replication and Meta-analysis Consortium (10) using a *P*-value threshold of 1. | | | | | | | | | | |  |
|  |  |  |  |  |  |  |  |  |  |  |  |
|  |  |  |  |  |  |  |  |  |  |  |  |
|  |  |  |  |  |  |  |  |  |  |  |  |
|  |  |  |  |  |  |  |  |  |  |  |  |

| \| **Supplementary Table 9.** Associations between polygenic score for Type 2 diabetes mellitus, Type 2 diabetes mellitus, and cognitive status, Health and Retirement Study, Wave 2010, African ancestry sample (n = 1889)^a^ \| \| \| \| \| \| \| \| \| \| \| \| \| --- \| --- \| --- \| --- \| --- \| --- \| --- \| --- \| --- \| --- \| --- \| --- \| \|  \| \| **Model 1: History of T2DM ~ T2DM polygenic score^b^** \| \| \| \| \| \| \| \| \| \| \| \|  \| \|  \|  \| **Overall sample  (n = 1862)** \| \| \| **CIND & Normal cognition sample (n = 1694)** \| \| \| \| **Dementia & Normal cognition sample (n = 1349)** \| \| \|  \| \|  \|  \| **OR (95 CI%)** \| \| \| **OR (95 CI%)** \| \| \| \| **OR (95 CI%)** \| \| \|  \| \| Crude \| \| 1.16 (1.04, 1.28) \| \| \| 1.15 (1.04, 1.29) \| \| \| \|  \| 1.16 (1.03, 1.31) \| \|  \| \| Adjusted (primary)^c^ \| \| 1.25 (1.10, 1.41) \| \| \| 1.27 (1.12, 1.45) \| \| \| \|  \| 1.29 (1.11, 1.50) \| \|  \| \| Improvement ꭓ^2 d^ \| \| 12.28 \| \| \| 13.14 \| \| \| \|  \| 11.40 \| \|  \| \| **Model 2: Cognitive Status ~ T2DM polygenic score** \| \| \| \| \| \| \| \| \| \| \| \|  \| \|  \|  \|  \| **CIND & Normal cognition sample**  **(n = 1694)** \| \| \| \| \| **Dementia & Normal cognition sample**  **(n = 1349)** \| \| \| \|  \| \|  \|  \|  \|  \| **OR** \| **(95 CI%)** \|  \|  \|  \| **OR** \| **(95 CI%)** \|  \|  \| \| **Total effect of T2DM polygenic score** \| \| \|  \| \| \| \| \| \| \| \| \|  \| \| Crude \| \| \|  \| 1.02 \| (0.92, 1.14) \|  \|  \|  \| 1.19 \| (1.01, 1.41) \|  \|  \| \| Adjusted (primary)^c^ \| \| \|  \| 1.07 \| (0.93, 1.22) \|  \|  \|  \| 1.05 \| (0.82, 1.36) \|  \|  \| \| Adjusted (health status)^e^ \| \| \|  \| 1.06 \| (0.92, 1.22) \|  \|  \|  \| 1.02 \| (0.79, 1.32) \|  \|  \| \| Adjusted (AD genetics)^f^ \| \| \|  \| 1.06 \| (0.92, 1.21) \|  \|  \|  \| 1.01 \| (0.78, 1.31) \|  \|  \| \| **Direct effect of T2DM polygenic score (adjusting for history of T2DM)** \| \| \| \| \| \| \| \|  \|  \|  \|  \|  \| \| Crude \| \| \|  \| 1.01 \| (0.91, 1.12) \|  \|  \|  \| 1.00 \| (0.88, 1.12) \|  \|  \| \| Adjusted (primary) \| \| \|  \| 1.08 \| (0.93, 1.25) \|  \|  \|  \| 1.06 \| (0.89, 1.26) \|  \|  \| \| Adjusted (health status) \| \| \|  \| 1.11 \| (0.95, 1.30) \|  \|  \|  \| 1.08 \| (0.90, 1.30) \|  \|  \| \| Adjusted (AD genetics) \| \| \|  \| 1.11 \| (0.95, 1.30) \|  \|  \|  \| 1.08 \| (0.90, 1.30) \|  \|  \| \| **Model 3: Cognitive Status ~ History of T2DM** \| \| \| \| \| \| \| \| \| \| \| \|  \| \|  \| **CIND & Normal cognition sample (n = 1694)** \| \| \| \| \|  \| **Dementia & Normal cognition sample (n = 1349)** \| \| \| \| \|  \| \|  \| **Logistic Regression** \| \| **Wald-type/ratio** \| \| ***P* for heterogeneity^g^** \|  \| **Logistic Regression** \| \| **Wald-type/ratio** \| \| ***P* for heterogeneity** \|  \| \|  \| **OR** \| **(95 CI%)** \| **OR** \| **(95 CI%)** \|  \| **OR** \| **(95 CI%)** \| **OR** \| **(95 CI%)** \|  \| \| Crude \| 1.45 \| (1.16, 1.82) \| 1.17 \| (0.56, 2.44) \| 0.58 \|  \| 1.27 \| (0.89, 1.80) \| 3.23 \| (1.08, 9.68) \| 0.11 \|  \| \| Adjusted (primary) \| 1.16 \| (0.91, 1.48) \| 1.30 \| (0.74, 2.30) \| 0.72 \|  \| 0.88 \| (0.56, 1.36) \| 1.23 \| (0.45, 3.33) \| 0.55 \|  \| \| Adjusted (health status) \| 1.18 \| (0.91, 1.51) \| 1.27 \| (0.72, 2.24) \| 0.82 \|  \| 0.85 \| (0.53, 1.35) \| 1.08 \| (0.39, 2.98) \| 0.67 \|  \| \| Adjusted (AD genetics) \| 1.17 \| (0.91, 1.51) \| 1.27 \| (0.72, 2.24) \| 0.79 \|  \| 0.85 \| (0.52, 1.35) \| 1.03 \| (0.37, 2.86) \| 0.74 \|  \| \| *Abbreviations: AD, Alzheimer's disease; APOE, Apolipoprotein E; BMI, body mass index; CI: confidence interval; CIND: cognitive impairment-non dementia; OR: odds ratio; T2DM: Type 2 diabetes mellitus.* \| \| \| \| \| \| \| \| \| \| \| \|  \| \| **Notes:** [a] All the values were based on results from multivariable logistic regression or Mendelian randomization analyses in each sample, in which "normal cognitive status" and "no diabetes history" were used as reference groups.  [b] The Type 2 diabetes mellitus polygenic score was created using weights from a genome-wide association study meta-analysis from the DIAbetes Genetics Replication and Meta-analysis Consortium (10) using a *P*-value threshold of 1. [c] Adjusted for age, sex, years of education, APOE-ε4 allele status, and five ancestry-specific principal component sets. [d] Calculated by 2*(log likelihood of full model - log likelihood of reduced model). By convention, statistics larger than 10 indicate a valid instrument. [e] Adjusted for smoking status, ever drink alcohol, history of stroke, hypertension, and BMI in addition to variables in [c]. [f] Adjusted for Alzheimer's disease polygenic score in addition to variables in [e]. The Alzheimer’s disease polygenic score was created using weights from a genome-wide association study meta-analysis from a 2019 GWAS by Kunkle et al. (22) using a P-value threshold of 0.01 and removing the APOE region (chr19: 45,384,477 to 45,432,606, build 37/hg 19) (21). [g] *P* value represents the statistical significance of the test of heterogeneity between logistic regression and Mendelian randomization. \| \| \| \| \| \| \| \| \| \| \| \|  \| \|  \| \|  \| \|  \| \|  \| \|  \| \|  \| |  |
| --- | --- | --- | --- | --- | --- | --- | --- | --- | --- | --- | --- | --- | --- | --- | --- | --- | --- | --- | --- | --- | --- | --- | --- | --- | --- | --- | --- | --- | --- | --- | --- | --- | --- | --- | --- | --- | --- | --- | --- | --- | --- | --- | --- | --- | --- | --- | --- | --- | --- | --- | --- | --- | --- | --- | --- | --- | --- | --- | --- | --- | --- | --- | --- | --- | --- | --- | --- | --- | --- | --- | --- | --- | --- | --- | --- | --- | --- | --- | --- | --- | --- | --- | --- | --- | --- | --- | --- | --- | --- | --- | --- | --- | --- | --- | --- | --- | --- | --- | --- | --- | --- | --- | --- | --- | --- | --- | --- | --- | --- | --- | --- | --- | --- | --- | --- | --- | --- | --- | --- | --- | --- | --- | --- | --- | --- | --- | --- | --- | --- | --- | --- | --- | --- | --- | --- | --- | --- | --- | --- | --- | --- | --- | --- | --- | --- | --- | --- | --- | --- | --- | --- | --- | --- | --- | --- | --- | --- | --- | --- | --- | --- | --- | --- | --- | --- | --- | --- | --- | --- | --- | --- | --- | --- | --- | --- | --- | --- | --- | --- | --- | --- | --- | --- | --- | --- | --- | --- | --- | --- | --- | --- | --- | --- | --- | --- | --- | --- | --- | --- | --- | --- | --- | --- | --- | --- | --- | --- | --- | --- | --- | --- | --- | --- | --- | --- | --- | --- | --- | --- | --- | --- | --- | --- | --- | --- | --- | --- | --- | --- | --- | --- | --- | --- | --- | --- | --- | --- | --- | --- | --- | --- | --- | --- | --- | --- | --- | --- | --- | --- | --- | --- | --- | --- | --- | --- | --- | --- | --- | --- | --- | --- | --- | --- | --- | --- | --- | --- | --- | --- | --- | --- | --- | --- | --- | --- | --- | --- | --- | --- | --- | --- | --- | --- | --- | --- | --- | --- | --- | --- | --- | --- | --- | --- | --- | --- | --- | --- | --- | --- | --- | --- | --- | --- | --- | --- | --- | --- | --- | --- | --- | --- | --- | --- | --- | --- | --- | --- | --- | --- | --- | --- | --- | --- | --- | --- | --- | --- | --- | --- | --- | --- | --- | --- | --- | --- | --- | --- | --- | --- | --- | --- | --- | --- | --- | --- | --- | --- | --- | --- | --- | --- | --- | --- | --- | --- | --- | --- | --- | --- | --- | --- | --- | --- | --- | --- | --- | --- | --- | --- | --- | --- | --- | --- | --- | --- | --- | --- | --- | --- | --- | --- | --- | --- | --- | --- | --- | --- | --- | --- | --- | --- | --- | --- | --- | --- |
|  |  |
|  |  |
|  |  |
|  |  |
|  |  |
|  |  |
|  |  |
